# Motivational predictors of children's involvement in out‐of‐school activities: An application of a football program

**DOI:** 10.1111/sms.14236

**Published:** 2022-09-22

**Authors:** Esben Elholm Madsen, Peter Krustrup, Tina Hansen, Charlotte Sandager Aggestrup, Nikos Ntoumanis, Carsten Hvid Larsen, Kristina Pfeffer, Andreas Ivarsson, Knud Ryom, Malte Nejst Larsen, Mads Madsen, Jeppe Elholm Madsen, Anne‐Marie Elbe

**Affiliations:** ^1^ Department of Sports Science and Clinical Biomechanics University of Southern Denmark Odense Denmark; ^2^ Department of Midwifery, Physiotherapy, Occupational Therapy and Psychomotor Therapy University College Copenhagen Copenhagen Denmark; ^3^ Faculty of Sport Science, Institute of Sport Psychology and Physical Education Leipzig University Leipzig Germany; ^4^ Danish Institute for Advanced Study (DIAS) University of Southern Denmark Odense Denmark; ^5^ Physical Medicine and Rehabilitation Research – Copenhagen, Department of Physiotherapy & Occupational Therapy Amager‐Hvidovre Hospital Copenhagen Denmark; ^6^ Danish Centre for Motivation and Behavior Science University of Southern Denmark Odense Denmark; ^7^ School of Health and Welfare Halmstad University Halmstad Sweden; ^8^ Department of Public Health, Section of Health Promotion and Global Health University of Aarhus Aarhus Denmark; ^9^ Department of Economics University of Copenhagen Copenhagen Denmark

**Keywords:** football, physical activity, self‐determination theory, theory of planned behavior, trans‐contextual model

## Abstract

The “11 for Health in Denmark” in‐school educational football program has shown to have numerous positive physiological and psychological effects in 10‐ to 12‐year‐old schoolchildren. A key part of the successful application of the program, however, has not yet been examined, namely the motivational processes underlying participation and behavioral changes. This study examined such motivational processes (i.e., autonomous motivation, beliefs, and intentions) using the trans‐contextual model (TCM) and investigated if the 11 for Health in Denmark program increased intentions to participate in physical activity (PA) outside of school in 10‐ to 12‐year‐old schoolchildren. Using a web‐based questionnaire, Danish‐speaking schoolchildren (*N* = 276 [boys, 50.4%]; *M*_age_ = 10.44, *SD* = 0.35) from three schools and seven classes completed TCM‐based questionnaires at three time‐points (weeks 0, 1, and 5) while participating in the 11 for Health in Denmark program. Single‐indicator structural equation modeling was performed to examine goodness‐of‐fit and parameter estimates. A path analysis using maximum likelihood estimation was used to test the direct and indirect effects of the TCM model. The results partly supported a mediation sequence, as we found significant direct effects in eight of 13 motivational variables (*β* = −0.25–0.83, *p* < 0.05), indirect effects in one of nine variables (*β* = 0.15, *p* < 0.01), but no effects with regard to PA behavior. Findings provide evidence for a motivational link between Danish‐speaking schoolchildren's autonomous and controlled motivation from in school to out of school, and may inform future interventions promoting motivation and participation in out of school PA.

## 1 INTRODUCTION

Epidemiological research has consistently demonstrated positive associations between physical activity (PA) and psychological, cognitive, and physical health outcomes in children and adolescents.^1^ Researchers have, therefore, sought to identify the psychological determinants of PA to inform the development of effective interventions to promote PA in adolescents. One of these determinants is motivation, which plays a key role in shaping engagement and attainment in PA and in physical education (PE).^2^ Motivational determinants are addressed in the multi‐theoretical trans‐contextual model (TCM; Hagger, Chatzisarantis, Culverhouse, and Biddle),^3^ according to which the promotion of autonomous motivation in classrooms will transfer toward autonomous motivation for activities in contexts outside of school, such as PA in leisure‐time.^4^ This trans‐contextual transfer is important, especially when considering that youth around the world often fail to achieve the recommended daily 60 min of moderate‐to‐vigorous PA.^5^ According to a report by the Danish Health Authority which in 2018 objectively measured PA levels in 1677 schoolchildren, this is also the case for Danish children between the age of 11–15 years, of whom only 26% meet the national PA guidelines.^6^

The 11 for Health in Denmark educational football concept (henceforth referred to as “the program”) was implemented in Danish schools in collaboration with the Danish Football Association (DBU) as a way to counter the inability to meet PA guidelines for children and has shown numerous positive physiological and psychological effects.^7^, ^8^, ^9^, ^10^, ^11^ Although the teachers who deliver the program are taught how to teach in an engaging manner, a key part of the program's successful application has not yet been examined; namely the motivational processes for participation and behavioral changes.^12^ Therefore, this study used the TCM framework to investigate the processes by which 10‐ to 12‐year‐old schoolchildren's perceived autonomy support and autonomous motivation toward the program‐predicted autonomous motivation, beliefs, intentions, and PA behavior outside of school. This line of research is unique as no previous research has applied the TCM in a football related PE intervention; further, it will be the first time that the TCM is tested in Danish‐speaking schoolchildren.

## 2 BACKGROUND

Increasing the number of PE lessons in school settings has shown a decrease in the percentage of schoolchildren with hypertension and prehypertension,^13^ improved physical status, and reduced prevalence of overweight, obesity, and cardiovascular risk.^14^ In response to such evidence, the World Health Organization (WHO) launched a global plan for PA for 2018–2030, in which whole school‐based policy initiatives are put forward as an essential component to creating more active children.^15^ This global plan aims to help schoolchildren reach the recommended 60 min/day of moderate‐to‐vigorous intensity aerobic PA across the week in order to achieve health benefits.^16^ Within a school context, PE holds a unique and advantageous position in being able to reach all children of an age cohort,^4^ and has been a useful pre‐existing network in which health promotion messages to support the adoption of health‐related PA behavior in young people can be promulgated.^17^ There are many ways to increase children and adolescent's daily PA; for instance, increasing PA in leisure‐time activity by being active in a leisure‐time sports club.^18^ Studies have shown that participating in leisure‐time sports increases the chances of meeting the WHO guidelines of undertaking at least 60 min/day of moderate‐to‐vigorous intensity.^19^ Relatively few studies have examined the effectiveness of school‐based PE interventions in promoting out‐of‐school PA,^20^ and seldom focusing on the motivational processes from a school to an out‐of‐school context.^21^ If the health benefits of PA are to be promoted, knowledge of the mechanisms of how in‐school interventions incorporating PA messages to children can motivate children to be active outside of school are therefore necessary.

### 2.1 The 11 for health in Denmark

The program is primarily designed for 10‐ to 12‐year‐old fifth‐grade schoolchildren and can be described as a health education program that takes place on the football pitch. The program combines health education and PA designed as small‐sided games or technical drills in small groups (e.g., visualizing healthy habits by dribbling a ball without hitting cones that represent cigarettes, etc.).^22^ When integrating the program into the school setting, it is required that the teachers participate in a cost‐free training course hosted by the DBU and held by researchers from the University of Southern Denmark. The training course lasts 2 days and its purpose is to ensure that the teachers deliver the program in an engaging and age‐, gender‐, and culture‐sensitive format.^7^ The training course uses a teaching manual with the intent to ensure clarity when “the program” is being delivered in the schools. The training course highly emphasizes autonomy‐supportive behaviors in the lessons, such as using informational rather than controlling language and providing positive feedback, but this style of teaching is not directly highlighted within the teaching manual. Within the literature, the program is described as a motivational school‐based concept,^8^, ^22^ but during the teacher training courses and within the teaching manual the motivational elements are described, very broadly. As an example of this the Appendix S1 of the teaching manual describes what constitutes a good teacher (i.e., show engagement, omit your views and values, let schoolchildren share their opinions, make eye contact, pay attention to schoolchildren's skills, avoid complex explanations, etc.). These descriptions are not based on theory or research but are described from researchers’ experience indirectly emphasizing autonomy‐supportive behaviors in the lessons, such as using informational rather than controlling language and providing positive feedback.

As shown in Table 1, the program consists of two weekly 45‐min sessions during an 11‐week period. The teacher decides which classes the two sessions should replace and one of the sessions often replaces PE, while the other replaces another session.^9^ The training focuses on delivering one of 10 health messages, ending with a final round‐up week (week 11). The sessions aim at a high level of PA for all those involved and include team exercises, but also group discussions on health topics. With few players per ball, the schoolchildren's level of involvement in the games are higher compared to normal team‐sport activities. Throughout the program, schoolchildren are encouraged within a PE school setting to engage in vigorous PA during out‐of‐school leisure‐time (i.e., riding their bike to school, playing football with family and/or friends, etc.).

**TABLE 1:** The “11 for Health in Denmark” program: Session activities, health messages, and topics (Madsen et al., 2020, p. 1789)

Week	“Play football” activity	“Plair fair” activity	Session topics
1	Warming up	Play football	Prepare for exercise and sport
2	Passing	Respect others	Respect and help others and avoid bullying
3	Goalkeeping	Be active	Walk, cycle, use the stairs in daily life
4	Dribbling	Avoid drugs, alcohol, and tobacco	Avoid unhealthy addictions
5	Controlling the ball	Control your weight	Control the quantity of food eaten
6	Defending	Wash your hands	Develop good hygiene
7	Trapping	Drink water	Drink water instead of soft drinks
8	Fitness training	Eat a balanced diet	Train and eat a varied diet
9	Overlapping	Keep fit	Do vigorous exercise
10	Shooting	Think positively	Have a positive mindset
11	Teamwork	Fair play	Review all health issues

A recent editorial in the *British Journal of Sports Medicine* by Thornton et al.,^23^ acknowledged the program for its wide‐ranging positive benefits on health and health knowledge. The authors praised the program for being both low cost and effective in improving health, and for offering a framework for educating children about lifelong health habits. Several studies targeting fifth‐grade schoolchildren completing the program have reported positive effects on the participants' physical fitness, cognitive performance, well‐being, enjoyment, and health knowledge. For instance, Lind et al.^7^ found that the program significantly improved psychomotor function and attention. Madsen et al.^9^ found a significant increase in physical well‐being. Ryom et al.^11^ found a positive impact on health knowledge, well‐being, fitness and high rates of enjoyment in ethnic minority schoolchildren. The evaluation of the program's intervention and implementation was also found to be positive from the involved teachers (Larsen, et al., 2018). Using a qualitative case study design, Madsen et al.^22^ found high adherence to the program primarily due to an autonomously supportive style of teaching, but also reported challenges of fitting an 11‐week program into a busy school schedule.

### 2.2 The trans‐contextual model

**FIGURE 1:**
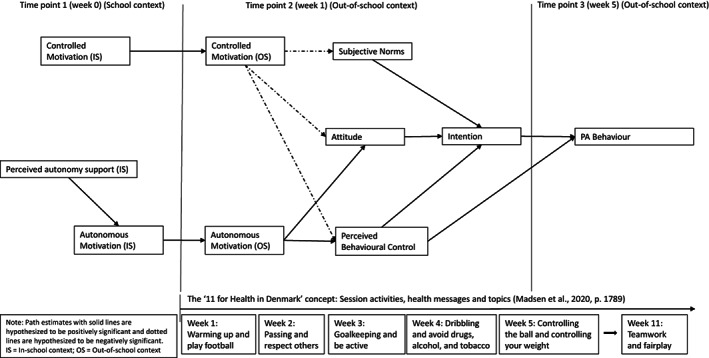
The original trans‐contextual model for the “11 for Health in Denmark” concept

The TCM outlines the motivational processes by which school children's autonomous motivation toward in‐school educational activities relate to autonomous motivation, intentions, and actual participations in related activities in an out‐of‐school context^24^ (please see Figure 1). As such, the model is an integrated approach, with a key focus on determinants in two contexts: PE and leisure‐time PA.^25^

In PE, the TCM explains the processes by which the PE teachers' support for autonomous motivation in the classroom promotes schoolchildren's motivation to engage in out‐of‐school PA. The strength of the TCM lies in the integration of different motivational theories, namely, self‐determination theory (SDT),^26^ the hierarchical model of motivation,^27^ and the theory of planned behavior.^28^ SDT focuses on how to engage the human self and emphasizes the social context and its ability to facilitate or thwart optimal motivation, and the extent to which behaviors are self‐determined or controlled, capturing both situational and personality‐related aspects of motivation.^26^ Vallerand^27^ adopted the premises from SDT and developed a model based on the relative level of generality of the motivational constructs, their antecedents, and their impact on outcomes. The theory of planned behavior^28^ can be described as an adopted social cognitive theory aimed at explaining intentional behavior. In this theory intentions are considered a motivational construct and represent the degree of planning and effort people are willing to invest in performing any future planned action or behavior. In the current study we used the TCM^29^ to investigate the processes by which 10‐ to 12‐year‐old schoolchildren's perceived autonomy support and autonomous motivation toward PE, based on the program, related to autonomous motivation, beliefs, intentions, and PA behavior with respect to the teacher‐set concept activities outside of school.

Most research on TCM originates from academic or school settings. In these settings the TCM has been used with various aims, such as to study motivational predictors of participation in‐school to out‐of‐school science learning activities in secondary‐school students,^21^ promoting secondary‐school students' participation in PA out‐of‐school contexts,^20^ and to test the effects of changes from a PE intervention in adolescents' outside of school moderate‐to‐vigorous PA.^25^ In these settings, the TCM assumes that PE teachers can foster autonomous motivation by adopting autonomy‐supportive actions and behaviors.^24^, ^29^ Face and content validity of the TCM constructs for a specific 57‐item 3‐wave web‐based questionnaire to be used in the program has been established in Danish schoolchildren.^12^

Applying the TCM to the program, is considered to help understand if school‐based football is motivating as stated in the literature,^8^, ^22^ and if the activities can enhance motivation and intentions to participate in out‐of‐school PA. Therefore, this study aimed to examine the processes by which 10‐ to 12‐year‐old schoolchildren's perceived autonomy support by their teacher and autonomous motivation related to autonomous motivation, beliefs, intentions, and actual behavior. The study also investigated if the program increases intentions to participate in active sports and/or vigorous PA outside of school.

## 3 STUDY HYPOTHESES

### 3.1 Direct effects of the trans‐contextual model (H_1_‐H_13_ )

For this study a specific set of hypotheses testing the TCM have been adopted and modified from Hagger and Hamilton^21^ (please see Appendix S1A for summary of hypothesized direct and indirect effects in the proposed TCM). The first hypothesis proposes that schoolchildren's perceived autonomy support by PE teachers will be positively related to autonomous motivation toward the program's activities in PE lessons (H_1_).

Drawing from Vallerand^27^ the second hypothesis propose that autonomous motivation toward the program's football activities in school will positively predict autonomous motivation toward similar activities in out‐of‐school (H_2_).

The third hypothesis proposes that controlled motivation toward the program's football activities will positively predict controlled motivation toward similar activities outside of school (H_3_).

According to the TCM, autonomous motivation toward activities in an out‐of‐school context will lead schoolchildren to strategically align their beliefs and intentions to engage in those activities in the future (i.e., participate in leisure‐time PA). As a contrast to this, controlled motivation is more likely to relate to beliefs that reflect pressure from significant others, captured by the subjective norms constructs.^21^ This implies that autonomous motivation toward out‐of‐school football activities will positively predict schoolchildren's attitudes (H_4_) and perceived behavioral control (H_5_). Because controlled motivation is more likely to relate to beliefs that reflect pressure from significant others^21^ we expect that controlled motivation toward out‐of‐school football activities will positively predict subjective norms (H_6_) and negatively predict attitude (H_7_) and perceived behavioral control (H_8_).

The last section of the TCM is related to the effects of beliefs on future behavior, consistent with the theory of planned behavior^28^ Specifically, attitudes (H_9_), subjective norms (H_10_), and perceived behavioral control (PBC) (H_11_) are proposed to positively predict intentions to engage in PA.^30^ Given that intentions are conceptualized as the most proximal predictor of PA behavior in studies with other schoolchildren,^31^ we expect intentions to positively predict schoolchildrens' PA behavior outside of school (H_12_). Assuming that PBC acts as a proxy for actual control over the behavior,^28^ we expect PBC to positively predict PA behavior (H_13_).

### 3.2 Indirect effects of the trans‐contextual model (H_14_‐H_22_ )

Using the TCM as a frame of reference, support for autonomy toward the program's football activities in school not only leads to autonomous motivation in the school context but also to autonomous motivation toward similar learning activities (such as: playing football, being active, riding your bike to school, eating a healthy diet, etc.).^9^, ^10^, ^32^ Therefore, perceived autonomous support, as the direct antecedent of in‐school autonomous motivation,^21^, ^33^ is hypothesized to positively predict autonomous motivation outside of school mediated by in‐school autonomous motivation (H_14_). Additionally, autonomous motivation toward in‐school football activities is hypothesized to positively predict intentions, mediated by autonomous motivation toward out‐of‐school football activities and attitudes (H_15_). Autonomous motivation toward in‐school football activities is hypothesized to positively predict intentions mediated by out‐of‐school controlled motivation and subjective norms (H_16_). Furthermore, autonomous motivation in‐school context is hypothesized to positively predict PA behavior mediated by autonomous motivation, attitude, and intentions (H_17_) and autonomous motivation, PBC, and intention (H_18_). Contrary to this, controlled motivation for in‐school football activities is expected to positively predict PA behavior mediated by out‐of‐school controlled motivation, subjective norm, and intentions (H_19_). Controlled motivation for out‐of‐school football activities is also expected to predict intention mediated by subjective norms (H_20_). Finally, we expect in‐school autonomous motivation to predict PA behavior mediated by PBC and intention (H_21_) and also out‐of‐school controlled motivation to predict PA behavior mediated by subjective norm and intention (H_22_).

## 4 MATERIALS AND METHODS

### 4.1 Procedure

Inspired by Hagger and Hamilton^21^ using a 5‐week prospective design in secondary school students, the participants completed the psychological measures after being introduced to the program (Time 1), after completing week 1 (Time 2), and again after completing week 5 of the intervention (Time 3). Schools from all over Denmark were sent regular invitations every 6 months from the DBU for their fifth‐grade classes to participate in the program in the period from August, 2016 ending in August, 2020. Schoolteachers voluntarily enrolled and participated in the program's teaching courses, which were geographically spread across the three largest cities in Denmark (Copenhagen, Aarhus, and Odense). For the present study, schoolteachers (6 males and 3 females) from nine different schools located in Copenhagen voluntarily signed up via DBU and participated in the program's teaching course in Copenhagen, Denmark in August 2019. After completing the two courses, seven out of nine schools agreed to participate in the study. Thereafter, information meetings were scheduled at the schools to inform them about the study and to obtain informed consent from the schoolchildren's parents. After the information meetings, the teachers were contacted to schedule the data collection. During data collection, from September to December, 2019, schoolchildren were informed by the researchers that responses reflected their opinions regarding the program, that their responses may be different from those of other children, and that there were no correct or incorrect answers. The questionnaires were distributed using the questionnaire tool Enalyzer.^34^ Questionnaires were completed and matched solely by the first author using names, date‐of‐birth, and school affiliation.

### 4.2 Measures

Details of the TCM questionnaire used in this study and validated by Madsen et al.,^12^ are summarized below and full details can be found in Appendix S1B. In the absence of actual observations on how teachers support autonomous motivation in the class, schoolchildren's perceived autonomy support by PE teachers were used.^21^ The Danish TCM questionnaire battery is available upon request from the corresponding author.

### 4.3 Time 1 (week 0)

Initially, personal information was collected (i.e., “name, age, grade, gender, teacher, and date‐of‐birth”). Hereafter 15 questions related to perceived autonomy support from each child's PE teacher^35^ (e.g., “I feel that my PE teacher provides me choices and options when doing the 11 for Health activities”), and eight questions related to the behavioral regulation items from the perceived locus of causality scale by Ryan and Connel^36^ (e.g., “I do 11 for Health because I want the PE teacher to think I'm a good student”). Autonomous and controlled forms of motivation for in‐school physical activities were rated on a Likert‐scale from 1 (very true) to 4 (not true at all). Perceived autonomy support from the PE teacher was rated on a Likert‐scale from 1 (strongly disagree) to 7 (strongly agree).

### 4.4 Time 2 (week 1)

Again, personal information was collected. Afterwards, 16 questions related to perceived locus of causality in a leisure‐time PA context were rated as in Time 1 and 18 questions related to the theory of planned behavior constructs.^28^ Three questions related to perceived behavioral control (e.g., “If I wanted to I could do active sports and/or vigorous physical activities in my leisure time in the next 5 weeks”) and four questions related to subjective norms (e.g., “Most people who are important to me think I should do active sports and/or vigorous physical activities during my leisure time for the next 5 weeks”). Three questions addressed intentions for a 5‐week PA participation, rated on a Likert‐scale from 1 (strongly disagree) to 7 (strongly agree) (e.g., “Now we would like to know about your intentions to train during your leisure time in the next 5 weeks outside school hours”). Five questions measured attitudes toward PA in leisure‐time, which were rated according to a set of 7‐point semantic differential scales covering boring‐interesting, unenjoyable‐enjoyable, bad‐good, useless‐useful, and harmful‐beneficial. The three questions related to perceived behavioral control addressing control over exercise during leisure‐time were rated on a Likert‐scale from 1 (very little control/strongly disagree) to 7 (complete control/strongly agree) (e.g., “If I wanted to I could do active sports and/or vigorous physical activities in my leisure time in the next 5 weeks”). The four questions related to subjective norms about PA in leisure time for the next 5 weeks were rated on a Likert‐scale from 1 (strongly disagree) to 7 (strongly agree) (e.g., “Most people who are important to me think I should do active sports and/or vigorous physical activities during my leisure time for the next 5 weeks”).

### 4.5 Time 3 (week 5)

Once again, personal information was collected. Hereafter, two questions related to engagement in active sports and/or vigorous PA (e.g., “I engaged in vigorous physical activity for 20 minutes at a time in the past five weeks at the following regularity”), were rated on a Likert scale from 1 (every day) to 6 (almost never). Schoolchildren's level of PA when participating in the program was computed as the mean of the two questions addressing engagement in active sports and/or vigorous PA.

### 4.6 ETHICS STATEMENT

The study was approved by the Regional Committees on Health Research Ethics for Copenhagen and Southern Denmark (H‐16026885).

### 4.7 Data analysis and statistics

Given the evidence supporting the TCM^24^, ^29^ researchers can make comparison when applying the model in other contexts. Traditionally a “frequentist approach” has been used to test effects in a proposed model, and an assumption within the frequentist approach is that there is one true population parameter that is fixed but unknown.^37^ However, the adoption of a “Bayesian approach” to analyze data from new tests allows researchers to incorporate such existing evidence into their analyses and, in doing so, provide more precise estimates in model tests.^38^ Therefore, we aimed to adopt a Bayesian path analysis on the pattern of effects among the constructs within the TCM into a test of the model in the context of PE. As the participants in Hagger and Hamilton^21^ were slightly older than our participants and the targeted classes were science education, we decided to deviate slightly from their analytical approach by estimating a single model predicated on informed priors from previous meta‐analysis.^29^

Initially, data were checked for normality and outliers using SPSS statistics, version 25. Since this study had a relatively small sample size, determining the distribution of all variables was crucial for choosing an appropriate statistical method. Therefore, a Shapiro–Wilk test was used to test whether the data were normally distributed and revealed that six variables were normally distributed (perceived autonomy support, controlled motivation in school and out‐of‐school, attitude, SN, and PBC). Three variables had negative skewness greater than one (autonomous motivation in school: W = 0.88, *p* = <0.001, intentions: W = 0.83, *p* = <0.001, and autonomous motivation out‐of‐school: W = 0.91, *p* = <0.001), indicating higher scores within autonomous motivation in school (Time 1), intentions (Time 2), and autonomous motivation out‐of‐school (Time 2). Finally, one variable revealed a positive skewness greater than one (amount of past PA behavior: W = 89, *p* = <0.001), indicating higher scores in perceived amount of PA behavior (Time 3). Hereafter, the descriptive statistics were calculated (please find mean and standard deviation at the bottom of Appendix S1B), then a correlation analysis was conducted among the study variables, and finally a path analysis of the predicted direct and indirect relationship hypothesized in the TCM was performed. The hypothesized relations among the variables in the TCM (summarized in Appendix S1A) were tested in a path analysis (with manifest variables, which carries the assumption that the measures are reliable manifestations of the constructs they represent) using Mplus software (version 8.0). The analysis was performed with the robust maximum likelihood estimator. Full information maximum likelihood, which is an estimation strategy providing parameter estimates, was used to handle missing data.

Traditional conventional fit indices^39^ were used to assess goodness of model fit, including chi‐square (χ^2^), the comparative fit index (CFI), Tucker–Lewis index (TLI), root mean square error of approximation (RMSEA), and standardized root mean square residual (SRMR) (i.e., χ^2^: *p* > 0.05, CFI ≥0.90, TLI ≥0.95, RMSEA <0.08, SRMR ≤0.05). For all specified parameters standardized regression coefficients were calculated together with a *p*‐value. A *p*‐value <0.05 was considered to indicate a statistically significant effect. We inspected non‐symmetric bootstrap confidence intervals (CI) to assess indirect effects. We considered the indirect effect to be statistically significant if the 95% CI did not include zero.

### 4.8 Participants

Participants were 276 Danish‐speaking fifth‐grade schoolchildren (*N* = 276, boys, *N* = 139, girls, *N* = 137; *M*_age_ = 10.44, *SD* = 0.35) from different schools (*N* = 3) and classes (*N* = 7).

## 5 RESULTS

The descriptive statistics, intercorrelations and internal consistency (Cronbach alpha) for all study variables at each time point are presented in Appendix S1B. All alpha values exceeded >0.70, except for the controlled motivation in PE construct (α = 0.53 at Time 1).

The schoolchildren's mean scores for autonomous motivation were higher than for controlled motivation both in‐school (3.42 vs. 2.99) and out‐of‐school (4.95 vs. 2.23). The mean scores for intentions (5.74), attitudes (5.65), subjective norms (5.05), PBC (5.47), and PA behavior were all rated positively by the schoolchildren based on the Likert‐scale from 1 (strongly disagree) to 7 (strongly agree).

**FIGURE 2:**
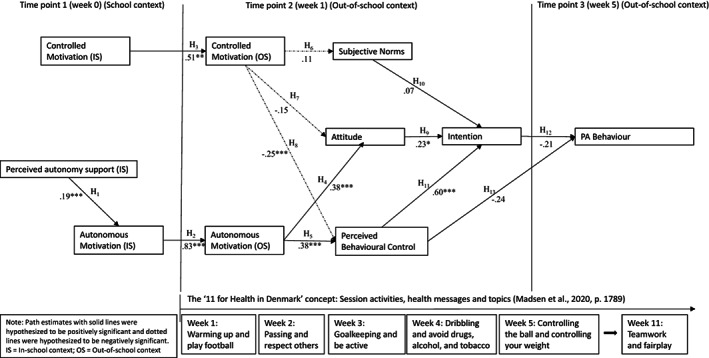
Hypothesis and direct effects of the original trans‐contextual model for the “11 for Health in Denmark” program

The fit indices obtained for the TCM suggested adequate model fit: χ^2^ (df) = 75.811 (26); *p* < 0.01, CFI = 0.911, TLI = 0.776, RMSEA = 0.083 (90% CI 0.062–0.105), SRMR = 0.089 (Figure 2).

Our results partly supported a mediation sequence proposed by the TCM, as we found positive significant direct effects in eight of 13 motivational variables (*β* = −0.25–0.83, *p* < 0.05), but no relationship with regards to promoting PA behavior. More specifically, we found a predicted, positive effect (*β* = 0.273, 95% CI = [0.04, 4.41]) from the schoolchildren's perceived autonomy support to autonomous motivation toward the program's activities in PE lessons, as hypothesized (H_1_). Autonomous motivation for the program's football activities positively predicted autonomous motivation toward similar activities in an out‐of‐school context (*β* = 0.824, 95% CI = [0.21, 3.86]), consistent with H_2_. Controlled motivation for the program's football activities in‐school predicted controlled motivation for similar out‐of‐school activities (*β* = 0.513, 95% CI = [0.16, 3.13]), as hypothesized (H_3_). Autonomous motivation toward out‐of‐school football activities positively predicted attitudes (*β* = 0.380, 95% CI = [0.07, 5.55]) (H_4_) and perceived behavioral control (β = 0.422, 95% CI = [0.07, 6.34]) (H_5_), as hypothesized. Controlled motivation toward out‐of‐school football activities did not predict subjective norms (*β* = 0.111, 95% CI = [0.08, 1.46]), contrary to the expectations (H_6_). Controlled out‐of‐school motivation did not negatively predict attitudes as expected (*β* = −0.146, 95% CI = [0.08, −1.93]) (H_7_), but did negatively predict perceived behavioral control (*β* = −0.248, 95% CI = [0.08, −3.21]) (H_8_). We found that attitudes (*β* = 0.227, 95% CI = [0.11, 2.05]) (H_9_) and perceived behavioral control (*β* = 0.595, 95% CI = [0.11, 5.26]) (H_11_) were positively related to intentions to engage in PA, but no effects were found with regard to subjective norms being related to intentions (*β* = 0.065, 95% CI = [0.06, 1.03]) (H_10_), as hypothesized. Lastly, we did not find any effect on the schoolchildren's intentions (*β* = −0.211, 95% CI = [0.14, −1.52]) (H_12_) and perceived behavioral control (*β* = −0.236, 95% CI = [0.18, −1.86]) (H_13_) to engage in PA behavior outside of school based on the program, as hypothesized.

Focusing on the indirect effects, we found that perceived autonomy support positively predicted autonomous motivation outside of school, mediated by in‐school autonomous motivation (ab = 0.153, 95% CI = [0.05, 3.1]) (H_14_). For the remaining proposed hypothesis (H_15_‐H_22_) we did not find any significant changes.

## 6 DISCUSSION

The present study applied the TCM to examine the processes by which 10‐ to 12‐year‐old schoolchildren's perceived autonomy support and autonomous motivation related to autonomous motivation, beliefs, intentions, and investigated if the program increased intentions to participate in active sports and/or vigorous PA. Our results partly supported a mediation sequence based on the TCM, as we found positive significant direct effects in 8 of 13 motivational variables (β = 0.19–0.82, *p* < 0.05), but no effects were found for promoting PA behavior.

Our results are thus somewhat consistent with previous prospective tests of the TCM,^40^ reviews and meta‐analysis^24^, ^29^ showing that the TCM can be a useful framework to understand the processes by which schoolchildrens' perceived autonomy support and autonomous motivation toward activities in an educational context are related to autonomous motivation toward activities in an out‐of‐school context.

Similarly to Schneider, Polet, Hassandra, Lintunen, Laukkanen, Hankonen, Hirvensalo, Tammelin, Törmäkangas and Hagger^41^ and to Barkoukis, Chatzisarantis and Hagger,^31^ we did not find support for the third phase of the TCM, namely that autonomous motivation in leisure time indirectly affected PA through intentions. Despite this, our study found support for multiple propositions of the TCM, such as direct effects of perceived autonomy support on autonomous motivation in PE, of autonomous motivation in PE on autonomous motivation in leisure time, and of autonomous motivation in leisure time on attitudes and PBC. In line with the findings of Barkoukis, Chatzisarantis and Hagger,^31^ but contrary to Schneider, Polet, Hassandra, Lintunen, Laukkanen, Hankonen, Hirvensalo, Tammelin, Törmäkangas and Hagger,^41^ we did not find that subjective norms predicted intentions.

Another reason for not finding support for the third phase of the TCM could be the “ceiling” effect in our PA behavior outcome variable. This ceiling effect could suggest that the scope for change was relatively small in terms of improving self‐reported PA behavior, as the schoolchildren considered themselves to be highly physically active. Also the limited time between the measures and that environmental and social variables other than those outlined by Madsen, Krustrup, Møller, Hansen, Larsen, Madsen, Hansen, Elbe and Larsen^22^ concerning the implementation facilitation of the program might have been more salient determinants of the schoolchildren's physical behavior than the psychological determinants identified within the TCM model. This is also supported by other studies stating more proximal factors that influence behavioral engagement, including motivational and cognitive constructs such as support for PA in leisure time by parents and peers, self‐efficacy and access to facilities and opportunities to participate.^42^, ^43^ This aspect underlines the multitude of potential determinants of leisure‐time PA engagement, such as access to sports clubs and organized PA in leisure time and the possibility of walking to school.^41^ Another explanation could be that people seem to be motivated by a variety of reasons at the same time, and that multiple motivational behavior regulations operate simultaneously, to create an overall motivational pattern of the individual.^44^ When looking into research concerning motivational profiles toward PE several studies have shown positive results outlining that different types of motivation co‐exist at the same time. As an example of this, a study of secondary school students in PE classes in Hong Kong identified four motivational profiles (i.e., non‐self‐determined, moderate controlled and low autonomous, high controlled and moderate autonomous, and self‐determined). The results revealed that students in different motivational profiles reported different affective experiences, underlying that students may undergo multiple affective experiences during PE. Another study also identified different motivational profiles (i.e., self‐determined and non‐self‐determined) and related those two to teacher autonomy support, basic psychological needs, exercise enjoyment, and level of PA in Brazilian adolescents. The results revealed that the self‐determined adolescents perceived greater teacher support, better fulfillment of basic psychological needs, showed greater enjoyment in PE and engaged in more PA.^45^ The results from these studies point to the fact that when investigating the motivational processes within a PE setting, such as with the program, it is important to consider that children have different and multiple reasons for engaging in the activities.

From a more methodological and theoretical stands our research expands the application of the TCM. By using prior knowledge from key effects in the model from Hagger and Chatzisarantis^29^ meta‐analysis, which are derived from multiple studies, we demonstrated how combining knowledge of the potential distribution of the model effects with our observed distribution leads to more precise estimates of relations among constructs in the model. As stated by Hagger and Hamilton^21^ we demonstrated this by the narrowing of the parameter credible intervals that represent the posterior distribution. Based on this we can state that our study contributed to further expanding the application of the TCM within a Danish physical education setting.

Football holds a great potential in increasing schoolchildren's PA levels,^46^ and helping children meet the required 60 min/day of moderate‐to‐vigorous intensity aerobic PA across the week. As children spend more time in school than anywhere other than home, the program applied within schools seems to be an excellent setting in which to offer quality physical activity education and possibilities for an active school day to a large number of children as recommended by the WHO.^47^ However, the program's training mostly focused on how to increase the physiological benefits for the schoolchildren, with limited, and mostly indirect, focus on how to increase motivation by using autonomy‐supportive techniques.^20^ The indirect focus on increasing motivation through autonomy‐supportive techniques are also somewhat addressed by Barkoukis, Chatzisarantis and Hagger,^31^ who speculated that their autonomy‐supportive intervention targeting PE teachers, was considered relatively brief and low in intensity. When compared to a study from Polet et al.,^20^involving 3 days' training with 3‐h sessions over a 5‐week period, Barkoukis, Chatzisarantis and Hagger's intervention seems brief as their intervention only comprised a series of three 1.5‐h seminars over a two‐week period. The explanations given by Barkoukis, Chatzisarantis and Hagger^31^ might also partly apply to our results, as our teaching only indirectly addressed autonomy‐supportive behavior, such as displaying patience and allowing the children to share their thoughts, be positive and curious, show enthusiasm and be appreciative, but did not have specific time‐frames addressing autonomy‐supportive techniques.

## 7 CONCLUSION

Based on the present results, it can be concluded that perceived autonomy support from the PE teachers applying the program was positively related to in school autonomous motivation toward the program. In‐school autonomous motivation toward the program was positively related to out‐of‐school autonomous motivation, and out‐of‐school autonomous motivation was positively related to attitudes and perceived behavioral control. In‐school controlled motivation toward the program was positively related to out‐of‐school controlled motivation and perceived behavioral control, but not to subjective norms and attitudes. Attitudes and perceived behavioral control were positively related to intentions, yet subjective norms were not related to intentions. No effects were found for promoting PA behavior. Findings provide evidence for a motivational link between Danish‐speaking schoolchildren's autonomous motivation for leisure time PA through a PE‐based football concept and may inform future interventions promoting motivation and participation in PA, both in Denmark and other countries.

## 8 LIMITATIONS

There are some limitations related to our study that are important to consider when making inferences. First, the Cronbach's alpha for the controlled motivation in PE construct (α = 0.53 at Time 1) was poor, when considering that the minimum acceptable value for Cronbach's alpha is 0.70. However, despite being the method most commonly used to assess internal consistency, Cronbach's alpha is considered to be sample‐dependent, which may lead to an overlook of a reliable measure or an adoption of an unreliable instruments.^48^ This need to be taken into consideration when interpreting our findings.

Second, as mentioned earlier our study solely relied on schoolchildren's' self‐reports, which needs to be considered when making inference. An advantage of using other more objective types of measures such as accelerometers in conjunction with the self‐reported scores might have strengthened our design, as other studies have shown that when measuring PA more objectively the statistical associations between motivational variables and PA levels might be lower than with the self‐reported PA scores.^49^ Finally, it is worth considering that we were not always capable of keeping the teachers out of the classroom when the schoolchildren answered the questionnaires. This may also have led to socially desirable responses.

## 9 RESEARCH PERSPECTIVES

The program holds great potential in increasing PA levels for children and could potentially be applied by other prominent governing bodies throughout the world. As such, other governing bodies of football such as the English FA, The United States Soccer Federation, the Royal Spanish Soccer federation etc. could develop similar programs or culturally adopt the program. However, before doing so the program can be improved in many ways, both regarding improving material and the mandatory teacher training courses. First the program now contains available online material, making teaching of the program easier and less time‐consuming for teachers (please see: Videos of the program). These videos could be used as inspiration in other countries. Second, the most effective way to support autonomous motivation in schools is for teachers to display autonomy‐supportive behaviors during PE lessons.^20^ Autonomy‐supportive teacher training programs should therefore be developed specifically for the program and implemented within the mandatory teacher training courses, both within Denmark and abroad. Normally, interventions like the program require specific training of teachers in adopting autonomy‐supportive behaviors (e.g., provision of choice, using informational rather than controlling language, providing positive feedback, and encouraging schoolchildren to take control of their learning) so that the teachers can implement these behaviors in their regular PE lessons.^41^, ^50^ As examples of this, Polet et al.,^20^ developed an interactive autonomy support teacher‐training program that aimed to familiarize PE teachers with techniques and strategies aimed at fostering autonomous motivation to promote secondary school students' PA participation. The teacher training program comprised a 2‐week, 12‐h training program in which the teachers received autonomy‐supportive training. The training was delivered by experienced teacher trainers as part of the teachers' regular in‐service training.^20^ Such autonomy‐supportive strategies and techniques should be implemented within the mandatory training courses to improve the program in the future. Such improvements could potentially form the basis of testing long‐terms effects of the program such as the effects of training teachers to be need‐supportive when teaching the program within Danish schools.

## CONFLICT OF INTEREST

The authors have no conflicts of interest to declare.

## Supporting information


Appendix S1

Supinfo S1

## Data Availability

The data that supports the findings of this study are available in the supplementary material of this article
