# Supplementary material for: Motivational predictors of children's involvement in out‐of‐school activities: An application of a football program
Source: Scand J Med Sci Sports. 2022 Sep 22;33(1):72–83. doi: 10.1111/sms.14236 (PMC10087348; doi:10.1111/sms.14236)
Supplement: Supplementary file 1 — Appendix S1 [file SMS-33-72-s001.docx]

**Appendix A.**

*Summary of hypothesised direct effects in the proposed trans-contextual model.*

|  | Independent Variable | Dependent Variable |  | Hypothesis |
| --- | --- | --- | --- | --- |
| H_1_ | Perceived autonomy support | Autonomous Motivation (IS) |  | Positive Effect |
| H_2_ | Autonomous Motivation (IS) | Autonomous motivation (OS) |  | Positive Effect |
| H_3_ | Controlled Motivation (IS) | Controlled Motivation (OS) |  | Positive Effect |
| H_4_ | Autonomous Motivation (OS) | Attitude |  | Positive Effect |
| H_5_ | Autonomous Motivation (OS) | PBC |  | Positive Effect |
| H_6_ | Controlled Motivation (OS) | SN |  | Positive Effect |
| H_7_ | Controlled Motivation (OS) | Attitude |  | Negative Effect |
| H_8_ | Controlled Motivation (OS) | PBC |  | Negative Effect |
| H_9_ | Attitude | Intention |  | Positive Effect |
| H_10_ | SN | Intention |  | Positive Effect |
| H_11_ | PBC | Intention |  | Positive Effect |
| H_12_ | Intention | PA behavior |  | Positive Effect |
| H_13_ | PBC | PA behavior |  | Positive Effect |
|  |  |  |  |  |

*Note*. IS = In-school context; OS = Out-of-school context; PAS = Perceived autonomy support; IS = In-school context; OS = Out-of-school context; ATT = Attitude; SN = Subjective norms; PBC = Perceived behavioural control; Physical activity behavior = PA behavior

**Appendix B.**

| Variables | 1 | 2 | 3 | 4 | 5 | 6 | 7 | 8 | 9 | 10 |
| --- | --- | --- | --- | --- | --- | --- | --- | --- | --- | --- |
| 1. Perceived Autonomy Support |  |  |  |  |  |  |  |  |  |  |
| 2. Controlled motivation (IS) | .26** |  |  |  |  |  |  |  |  |  |
| 3. Autonomous motivation (IS) | .41** | .61** |  |  |  |  |  |  |  |  |
| 4. Controlled motivation (OS) | .03 | .24** | .12 |  |  |  |  |  |  |  |
| 5. Autonomous motivation (OS) | .03 | .14 | .30** | .30** |  |  |  |  |  |  |
| 6. Intention | .21** | .15 | .30** | -.01 | .53** |  |  |  |  |  |
| 7. Attitude | .20** | -.01 | .27** | .13 | .43** | .62** |  |  |  |  |
| 8. SN | .10 | .11 | .17* | .20** | .28** | .38** | .47** |  |  |  |
| 9. PBC | .29** | .07 | .29** | -.05 | .44** | .71** | .72** | .42** |  |  |
| 10. PA. Behaviour | -.14 | -.06 | -.29** | -.14 | -.16 | -.38** | -.31** | -.18 | -.37** |  |
| Cronbach’s Alpha | .71 | .53 | .79 | .81 | .89 | .87 | .92 | .71 | .76 | .91 |
| Mean | 4.80 | 2.99 | 3.42 | 2.23 | 4.95 | 5.74 | 5.65 | 5.05 | 5.47 | 1.93 |
| Standard Deviation | .82 | .55 | .58 | 1.14 | 1.14 | 1.24 | 1.12 | 1.19 | 1.15 | 1.01 |

*Descriptive statistics, internal reliability and intercorrelations between the trans-contextual model variables (N=257)*

*Note*. IS = In-school context; OS = Out-of-school context; PAS = Perceived autonomy support; IS = In-school context; OS = Out-of-school context; ATT = Attitude; SN = Subjective norms; PBC = Perceived behavioural control; Physical activity behavior = PA behavior.

* *p* <.05 ** *p* <.01*** *p* <.001
